# Deficient gait function despite effect index of the Western Ontario and McMaster university osteoarthritis index score considered cured one year after bilateral total knee arthroplasty

**DOI:** 10.1186/s12891-024-07348-7

**Published:** 2024-03-23

**Authors:** Ruipeng Zhao, Xiaochun Wei, Shuai Hu, Yixuan Zhang, Hongru Wu, Pengcui Li, Yu Zhao

**Affiliations:** 1 Department of Orthopaedics, Shanxi Key Laboratory of Bone and Soft Tissue Injury Repair, The Second Hospital of Shanxi Medical University, 382 Wuyi Road, Taiyuan, 030001 Shanxi China; 2 Shanxi Institute of Sports Science, Taiyuan, 030001 Shanxi China

**Keywords:** Osteoarthritis, Total knee arthroplasty, Gait analysis, Self-reported function, Correlation analysis

## Abstract

**Background:**

To clarify the value of gait analysis and its consistency with traditional scoring scales for the evaluation of knee joint function after total knee arthroplasty (TKA).

**Methods:**

This study included 25 patients with knee osteoarthritis (KOA) who underwent bilateral TKA, and 25 conditionally matched healthy individuals, categorised into the experimental and control groups, respectively. Patients in the experimental group underwent gait analysis and Western Ontario and McMaster University Osteoarthritis Index (WOMAC) evaluation before and 1 year after TKA. Weight-bearing balance and walking stability were assessed using discrete trends of relevant gait indicators. Pearson’s correlation analysis was performed on the gait and WOMAC score data of the experimental group before and after TKA.

**Results:**

One year after TKA, patients’ gait indices (except gait cycle) were significantly better than before surgery, but significantly worse than that of the control group (*P* < 0.01). The shape of patients’ plantar pressure curves did not return to normal. Additionally, the discrete trend of related gait indicators reflecting weight-bearing balance and walking stability were smaller than before TKA, but still greater than that of the control group. The WOMAC scores of patients 1 year after TKA were significantly lower than those before TKA (*P* < 0.001), and the efficacy index was > 80%. The WOMAC scores and gait analysis results were significantly correlated before TKA (*P* < 0.05).

**Conclusions:**

Gait analysis should be used in conjunction with scoring scales to assess joint functions.

**Supplementary Information:**

The online version contains supplementary material available at 10.1186/s12891-024-07348-7.

## Introduction

Knee osteoarthritis (KOA) is a common, chronic, degenerative joint disease in the middle-aged and older adults. The disease affects millions of individuals, with pain, deformation, and limited mobility resulting in major healthcare costs [1, 2]. Total knee arthroplasty (TKA) is the most effective treatment for end-stage KOA. In the United States, > 500,000 TKAs are performed annually to alleviate the pain associated with OA [3, 4], and joint functional recovery after TKA is primarily evaluated using scoring scales. These rating scales mainly rely on the patient’s feelings, naked-eye observations of the medical staff, and some physical examinations, which are highly subjective and sometimes cannot reflect the real disease situation [5, 6]. Additionally, long-term pain has plagued patients with KOA, and many patients fear pain.

Since, patients with KOA demonstrate pain sensitisation and hypervigilance to pain [7, 8], they perceive pain relief as their primary purpose for undergoing TKA. Self-reported measures of function are largely influenced by pain, and if the perceived pain is greatly reduced, the function assessment may be overestimated or confused with improved function, rather than reduced pain [9]. Therefore, a method or technique that objectively and accurately evaluates the knee joint function is important. Gait analysis can measure the lower limb’s spatiotemporal parameters by dynamically examining the angle changes of joint centres. Additionally, it is suitable to apply gait analysis to assess knee joint function post-TKA [10, 11]. Therefore, this study aimed to clarify the value of gait analysis for evaluating knee joint function, and its consistency with traditional scoring scales for evaluating function after TKA. We hypothesised that while knee function would significantly improve 1 year after TKA, gait function would not return to normal in patients with satisfactory self-reports.

## Materials and methods

This study was approved by the Ethics Committee of The Second Hospital of Shanxi Medical University (NO.20,200,860). All participants provided informed consent, following the Declaration of Helsinki.

**Fig. 1:**
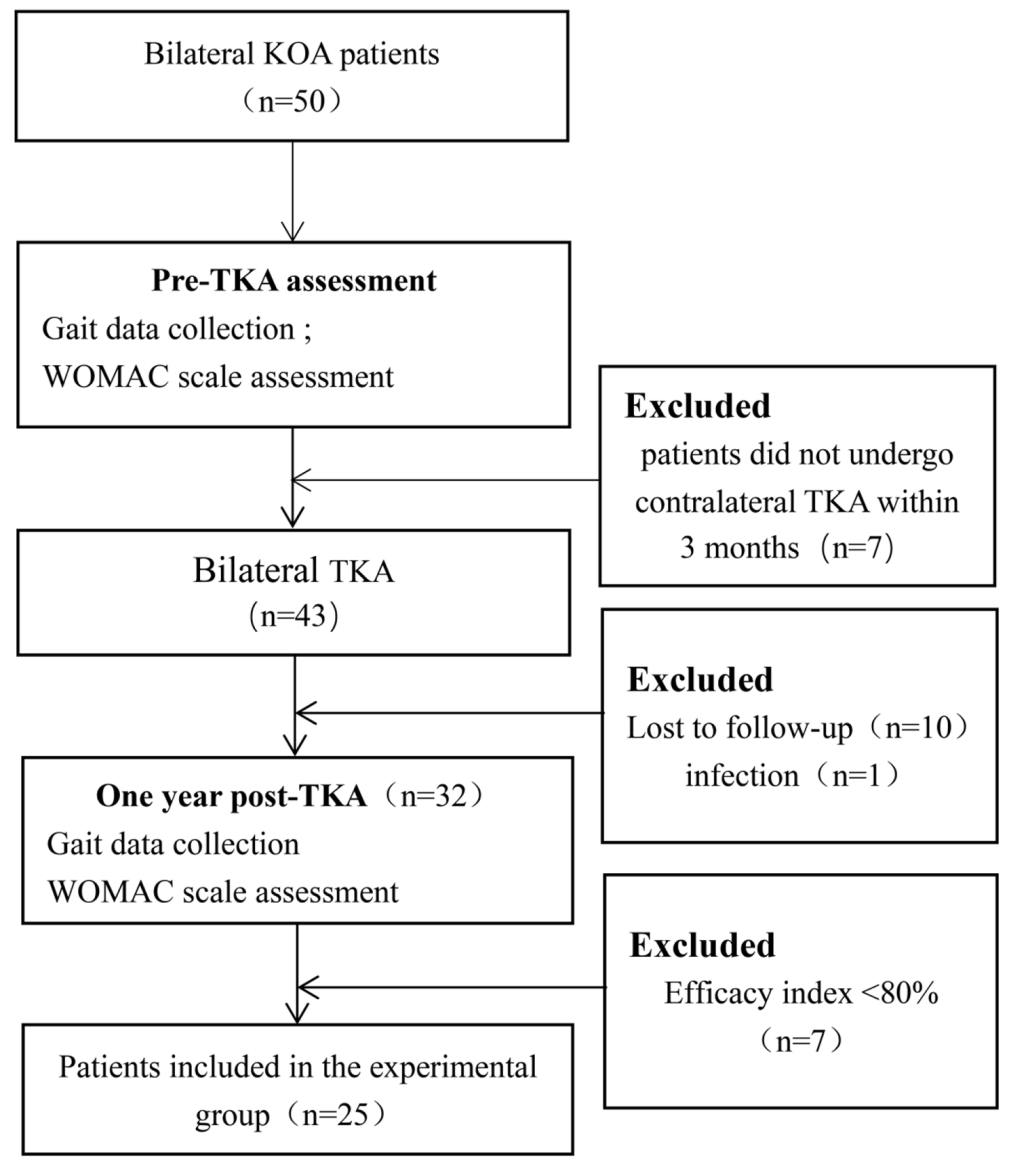
Flowchart of included patients with bilateral knee osteoarthritis, test procedures, excluded patients, and patients completing 1-year of follow-up

Twenty-five patients with KOA who underwent bilateral TKA in the Joint Surgery Department of The Second Hospital of Shanxi Medical University between July and December 2020 were selected as the experimental group. The inclusion criteria were as follows: a)end-stage bilateral KOA diagnosed according to the American College of Rheumatology [12] and confirmed as grade III or IV following the Kellgren–Lawrence system [13]; b) the patient was ready to undergo bilateral TKA simultaneously, and c) thea stage interval within 3 months. The exclusion criteria were as follows: (a) mental and psychiatric disorders affecting normal walking; (b) concurrent heart, lung, and brain disease affecting walking; (c) history of lower extremity and spine surgery; (d) rheumatoid arthritis; and (e) knee joint function score that did not meet the established standards 1 year after TKA. The inclusion criteria of the control group were as follows: (a) baseline data (including age, height, weight, and sex) were not significantly different from the experimental group; (b) the WOMAC score was 0; (c) the line of force in the lower extremities was straight; and (d) no injuries or surgery occurred on the lower extremities or spine. Finally, 25 patients were included in the experimental group (Fig. 1), and 25 conditionally matched healthy individuals were recruited to the control group.

### TKA surgery and post‑operative rehabilitation

All TKA surgeries were performed by the same surgical team. Posterior cruciate ligament retaining prostheses (*n* = 40) and posterior stabilised prostheses (*n* = 10) were used in the surgery. All patients underwent relatively consistent rehabilitation programs after TKA.

### Gait data collection

The gait pressure distribution flat panel test system (Footscan 2 m HE; RSscan International NV Belgium) was placed on a flat, hard surface. The connection of the two codamotion cameras using a three-dimensional dynamic joint motion capture system (2CX1; Charnwood dynamics limited; Britain) was perpendicular to the long axis of the footscan, wherein the distance was guaranteed to be 7 m. The instruments were calibrated separately [14]. Participants were informed of the purpose of the examination and precautions; they completed the test barefoot, fully exposed to their lower limbs and waist, and wore the relevant examination kits during the examination (Supplementary Fig. 1). Complete gait was collected three times per examiner, and the average value of the three records was used for the gait index data [15]. Gait spatiotemporal parameters (velocity, cadence, step length, stride length, step time, gait cycle, total stance time, double stance time, and single stance time) and knee joint motion parameters (range of motion of the knee joint, maximum flexion angle, and minimum extension angle) were collected. The experimental group underwent gait examination before and 1 year after TKA. We further noted shape changes in the plantar pressure curve of dynamic walking.

### Weight-bearing balance and walking stability assessment

To reduce the influence of errors and individual differences, weight-bearing balance (static standing bipedal weight bearing, dynamic walking bipedal pressure) and walking stability (step length and step time) gait indicators were expressed using ratios (left: right lower extremities). This was measured by the size of the indicator that described the discrete trend; the larger the value, the higher the degree of dispersion, and the poorer the balance and stability [16, 17].

### Scoring scale

Knee joint function was evaluated pre-TKA and 1-year post-TKA in the experimental group using the Western Ontario and McMaster University Osteoarthritis Index (WOMAC) scale [18]. On this scale, the higher the score, the poorer the knee joint function. Effect assessment 1 year after TKA was determined using the nimodipine calculation method; i.e. effect index = (the score before treatment – the score after treatment)/the score before treatment*100%. An effect index ≥ 80% was considered corresponding to cured. Effect indices ≥ 50% and < 80% were considered markedly effective, ≥ 25% and < 50% were considered effective, and < 25% was considered invalid [19]. Patients with an effect index ≥ 80% were considered to meet the purpose of the experimental design and were eventually included in the experimental group.

### Correlation analysis

Self-reported measures of function are largely influenced by pain; therefore, correlation analyses between the pain subitem and the remaining items of the WOMAC score were performed pre-TKA and 1 year post-TKA [20]. Correlation analyses between gait analysis results and WOMAC scores of patients in the experimental group were performed before and 1 year after TKA.

### Statistical analysis

SPSS v13.0 statistical software was used to analyse the collected data. Measurement data were expressed as mean ± standard deviation (SD). Gait data of the experimental and control groups were compared using an unpaired two-group t-test; gait data of the experimental group before and after TKA were compared using a paired two-group t-test. Pearson’s correlation analysis (between gait data, WOMAC data, and WOMAC subitems of patients in the experimental group) was performed before and after TKA. Statistical significance was set at *P* < 0.05. The discrete trend indicators used to evaluate weight balance and walking stability were variance and SD.

## Results

There were no significant differences in the baseline data between the experimental and control groups (Table 1).

**Table 1:** Comparison of the general information between experimental and control groups (*n* = 25)

Parameter	Experimental group	Control group	P value
Gender (Male/ Female)	10/15	12/13	0.776
Age (Year)	67.12 ± 6.04	64.04 ± 9.49	0.186
Height (cm)	164.28 ± 6.93	163.92 ± 6.37	0.852
Weight (Kg)	71 ± 7.62	67.64 ± 8.77	0.136
Follow-up time (months)	12.08 ± 1.2		

### Gait analysis

One year after TKA, patients’ gait indices — excluding gait cycle — were significantly better than before TKA; however, these remained significantly worse than that of the control group. In the experimental group, velocity, cadence, step length, stride length, and single stance time post-TKA increased significantly compared with the pre-operative values; still, these values remained significantly lower than those of the control group (*P* < 0.01, Table 2). By contrast, the step time, total stance, and double stance times post-TKA in the experimental group reduced significantly compared with the pre-operative values; nonetheless, they were still significantly greater than those of the control group (*P* < 0.01, Table 2). There was no significant decrease in the post-operative gait cycle compared with the pre-operative values (*P* = 0.543, Table 2). The range of motion and maximum flexion degree of the left and right knee joints in the experimental group post-TKA increased significantly compared with the pre-operative values; yet, they were still significantly lower than in the control group (*P* < 0.001, Table 2). By contrast, the minimum degree of knee extension after TKA was reduced compared with the values before TKA; however, it was still significantly greater than in the control group (*P* < 0.001, Table 2).

**Table 2:** Comparison of gait parameters between experimental and control groups (*n* = 25)

Gait parameters	Pre-TKA	Post-TKA	Control group	P value
Velocity (m/s)	0.53 ± 0.19	0.84 ± 0.15	1.05 ± 0.20	*#&
Cadence (step/min)	87.87 ± 12.25	97.19 ± 7.64	111.86 ± 16.33	*#&
Step length (cm)	34.16 ± 9.28	46.22 ± 6.28	51.71 ± 4.88	*#&
Stride length (cm)	65.00 ± 19.81	90.31 ± 11.62	102.78 ± 9.00	*#&
Step time (ms)	695.92 ± 95.49	621.09 ± 47.56	546.15 ± 68.76	*#&
Gait cycle (ms)	1303.89 ± 166.67	1283.05 ± 78.80	1081.02 ± 121.90	*#
Total stance time (%)	75 ± 6.74	67 ± 5.27	64 ± 4.80	*#&
Double stance time (%)	45 ± 10.50	37 ± 3.58	31 ± 2.52	*#&
Single stance time (%)	28 ± 4.33	31 ± 2.2	33 ± 1.26	*#&
left knee range of motion (°)	38.19 ± 6.64	52.74 ± 4.11	62.75 ± 3.99	*#&
Maximum left knee flexion (°)	53.10 ± 5.08	61.34 ± 3.34	65.93 ± 3.40	*#&
Minimum left knee extension (°)	15.23 ± 4.24	8.91 ± 2.03	3.27 ± 1.52	*#&
right knee range of motion (°)	35.36 ± 6.73	53.83 ± 4.35	61.7 ± 3.08	*#&
Maximum right knee flexion (°)	52.27 ± 5.38	61.82 ± 3.07	65.29 ± 3.18	*#&
Minimum right knee extension (°)	17.15 ± 4.16	8.14 ± 2.22	3.14 ± 1.41	*#&

Data are presented as Mean ± SD

% Represents the proportion in the Gait cycle

* indicates significant differences between the pre-TKA and control groups (*P* < 0.01)

# indicates significant differences between the post-TKA and control groups (*P* < 0.01)

& indicates significant differences between the pre -and post-TKA groups (*P* < 0.01)

**Fig. 2:**
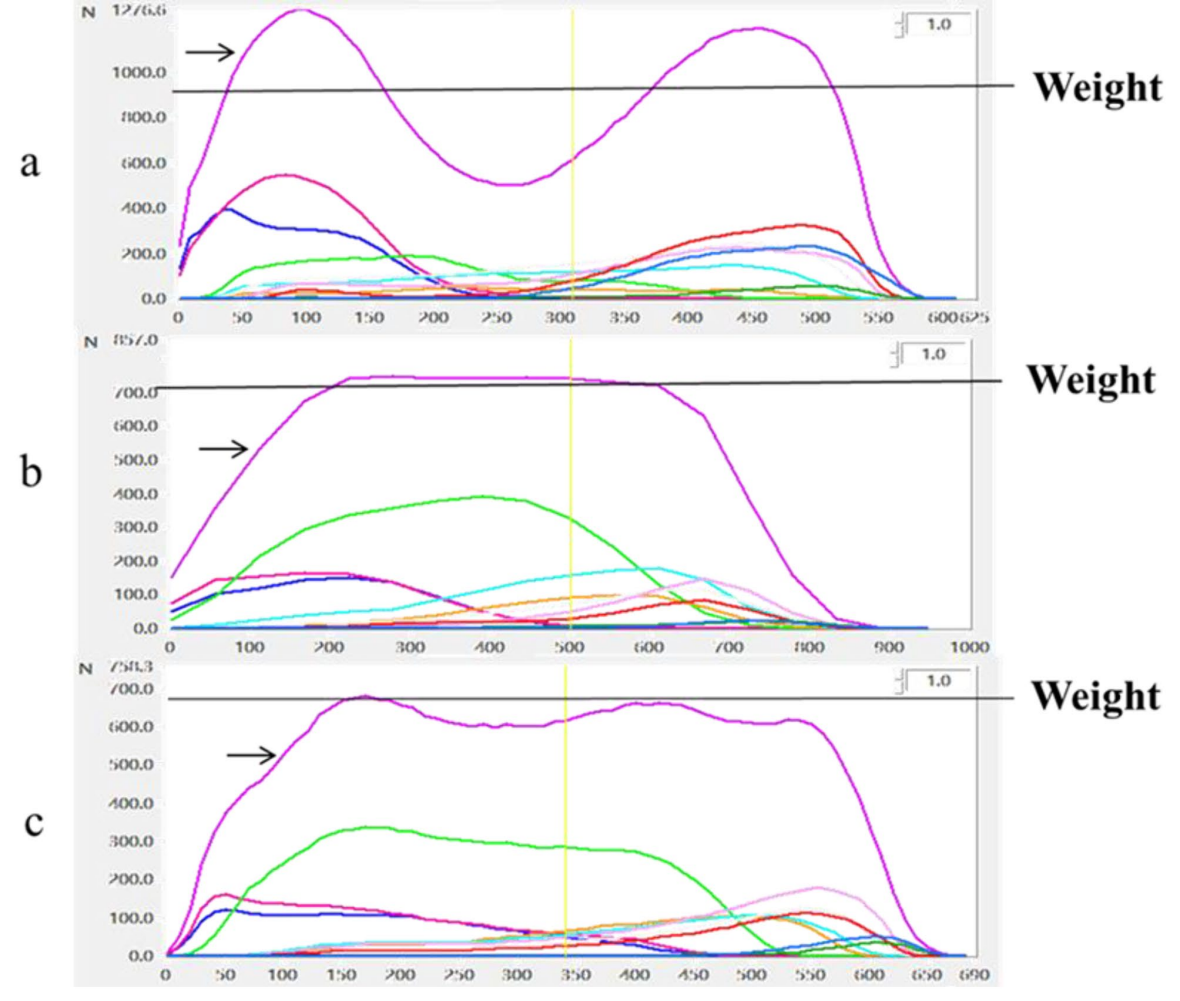
The dynamic walking plantar pressure curve. (a) Control group (b) Pre-TKA group (c) Post-TKA group. The abscissa is the total stance time ( ms ). The vertical axis is the dynamic walking ground return force ( Newton ). The pink curve (black arrow) represents the total plantar pressure. The colours below the pink curve show the pressure on different plantar areas. The black horizontal lines represent weight. The yellow vertical line represents half of the stance time

The plantar pressure curve of dynamic walking significantly differed between the control and experimental groups pre- and post-TKA. The plantar pressure curve of the control group had a double peak ‘m’ shape; the maximum pressure value (wave crest) was significantly greater than the weight, whereas the minimum pressure value (trough) was significantly less than weight. Pre-TKA, patients in the experimental group had a single-peak ‘n’ shape; the maximum pressure value (wave crest) was close to the body weight. The shape of the post-TKA plantar pressure curve did not return to normal, and the peak pressure was close to the weight (Fig. 2).

### Weight-bearing balance and walking stability assessment

One year after TKA, the discrete trend of related gait indicators was smaller than before TKA; still, this was greater than in the control group. The variance and SD of weight-bearing balance gait indicators (static standing bipedal weight-bearing and dynamic walking bipedal pressure) and the walking stability gait indicators (step length and step time) in the experimental group after TKA were significantly reduced compared with those before TKA; however, they were still significantly greater than those in the control group (Table 3).

**Table 3:** Discrete trend of weight-bearing balance and walking stability gait indicators in experimental and control group (*n* = 25)

	Variance			Standard deviation		
	Pre-TKA	Post-TKA	Control group	Pre-TKA	Post-TKA	Control group
static standing bipedal weight bearing	0.186	0.042	0.009	0.431	0.204	0.097
dynamic walking bipedal pressure	0.019	0.009	0.001	0.113	0.097	0.038
Step length	0.058	0.006	0.001	0.241	0.077	0.035
Step time	0.019	0.007	0.001	0.138	0.081	0.031

### WOMAC score

The WOMAC total and sub-item scores of patients 1-year post-TKA were significantly lower than those pre-TKA (*P* < 0.001, Table 4). The effect index of both the sub-items and total items was > 80% (Table 4).

**Table 4:** WOMAC score of patients in the experimental group at Pre-TKA and Post-TKA (*n* = 25)

	Pain (points)	Stiffness (points)	Daily living function (points)	Full score (points)
Pre-TKA	9.76 ± 1.70	2.64 ± 0.84	35.36 ± 6.00	47.76 ± 8.11
Post-TKA	1.12 ± 0.59	0.44 ± 0.49	4.32 ± 1.22	5.88 ± 1.82
Efficacy index(%)	88 ± 6.26	80 ± 28.56	87 ± 3.33	88 ± 3.76
P value	< 0.001	< 0.001	< 0.001	< 0.001

Data are presented as Mean ± SD

Efficacy index = (pre-TKA score - post-TKA score)/ pre-TKA score × 100%

### Pearson correlation analysis

**Fig. 3:**
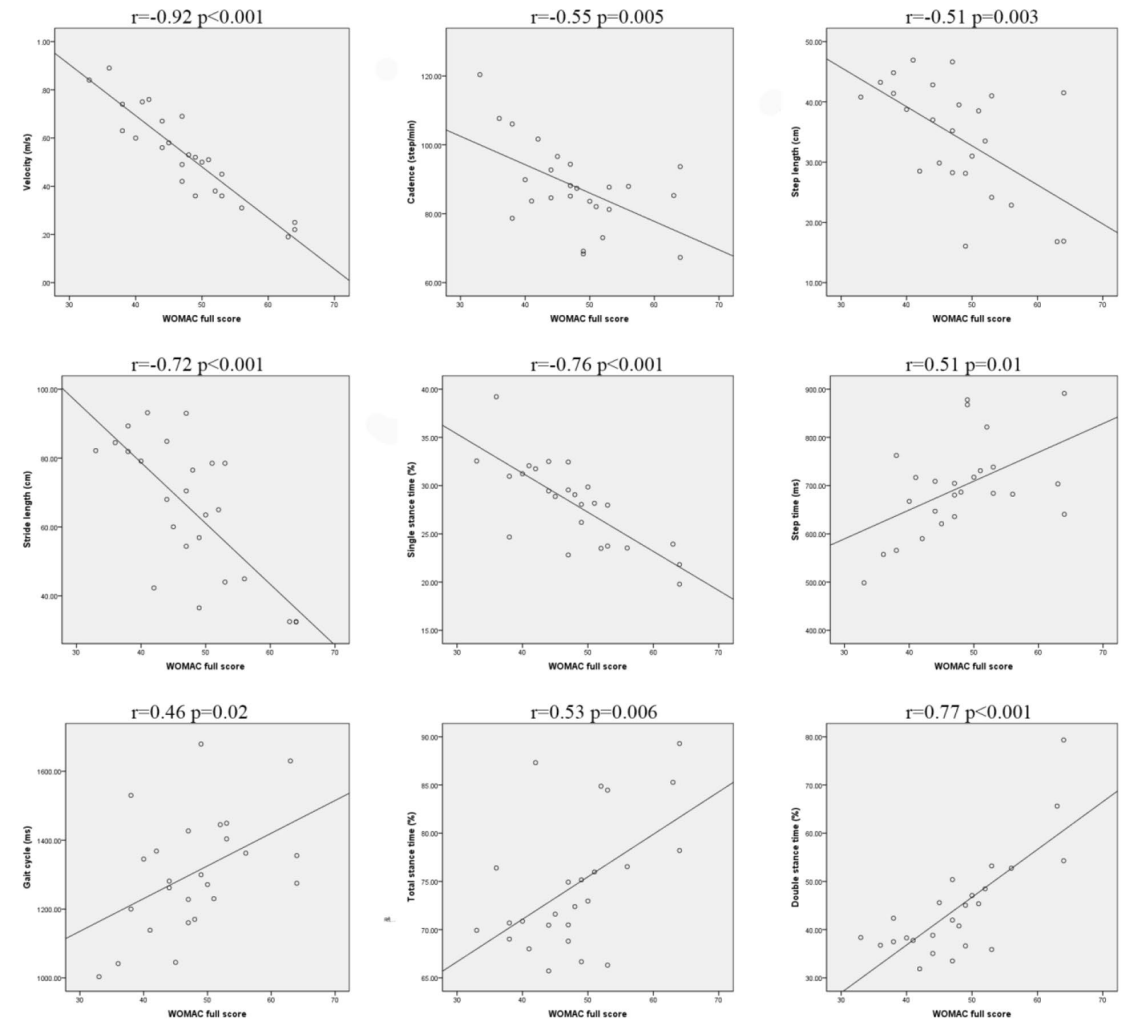
Pearson correlation analysis of WOMAC full score and gait analysis in the experimental group before TKA. There was significant correlation between the WOMAC full score and gait analysis parameters before TKA. There was a significant negative correlation between WOMAC full score and velocity, cadence, step length, stride length, and single stance time in gait analysis (*P* < 0.01). There was a significant positive correlation between step time, gait cycle, total stance time, double stance time, and sub-item and WOMAC full scores (*P* < 0.05)

Significant positive correlations were observed between the pain subscale and remaining items of the WOMAC score before (*P* < 0.001, Table 5) and after surgery (*P* < 0.05, Table 5). Pearson correlation analysis demonstrated a significant correlation between the WOMAC score and gait analysis pre-TKA (*P* < 0.05, Table 6; Fig. 3). There was a significant negative correlation between the pain score, daily living function score, and total WOMAC score; and velocity, cadence, step length, stride length, and single stance time in gait analysis (*P* < 0.01, Table 6; Fig. 3). There was a significant positive correlation between step time, gait cycle, total stance time, double stance time, and sub-item and total WOMAC scores (*P* < 0.05, Table 6; Fig. 3). The stiffness score in the WOMAC correlated negatively with the velocity, stride length, and single stance time in the gait analysis (*P* < 0.05, Table 6), and correlated positively with the total stance and double-stance times (*P* < 0.05, Table 6). There was no significant correlation between the WOMAC score and gait analysis 1 year after TKA (*P* > 0.05, Supplementary Table 1, Supplementary Fig. 2).

**Table 5:** Pearson correlation analysis between pain subitem and remaining items of the WOMAC score (*n* = 25)

	Pre-TKA						Post-TKA					
	Stiffness		Daily living function		Full score		Stiffness		Daily living function		Full score	
	r	p	r	p	r	p	r	p	r	p	r	p
Pain	0.66	< 0.001	0.84	< 0.001	0.90	< 0.001	0.37	0.071	0.39	0.050	0.69	< 0.001

**Table 6:** Pearson correlation analysis of WOMAC score and gait analysis in the experimental group before TKA (*n* = 25)

	Pain		Stiffness		Daily living function		Full score	
	r	p	r	p	r	p	r	p
Velocity	-0.84	< 0.001	-0.66	< 0.001	-0.91	< 0.001	-0.92	< 0.001
Cadence	-0.56	0.003	-0.17	0.410	-0.53	0.004	-0.55	0.005
Step length	-0.57	0.003	-0.24	0.259	-0.57	0.003	-0.51	0.003
Stride length	-0.67	< 0.001	-0.49	0.014	-0.72	< 0.001	-0.72	< 0.001
Step time	0.54	0.006	0.11	0.598	0.52	0.008	0.51	0.010
Gait cycle	0.45	0.026	0.16	0.438	0.48	0.016	0.46	0.020
Total stance time	0.47	0.017	0.44	0.027	0.52	0.008	0.53	0.006
Double stance time	0.63	0.001	0.73	< 0.001	0.75	< 0.001	0.77	< 0.001
Single stance time	-0.69	< 0.001	-0.47	0.018	-0.77	< 0.001	-0.76	< 0.001

## Discussion

Although 20–30% of patients report a persistent disability, limited function, reduced quality of life, diminished working capacity, and gait deviations post-TKA, TKA remains the most effective treatment for end-stage KOA [21–23]. One year of follow-up was selected as patients recovering from bilateral TKA typically plateau in strength and functional gains at this time point [24, 25]. This study confirmed that patients’ self-perception and gait function significantly improved after TKA; therefore, velocity, a gait indicator that reflects the comprehensive ability to walk, increased significantly one year after TKA. In other words, patients’ overall walking function improved significantly. Stance time can accurately reflect the stability and fluency of a patient’s walk and indicates pain sensitivity. When the total stance time (especially the double support time) is too long, it indicates that the walking stability of the patient is poor, not smooth, and the patient is ‘stuck’. Conversely, when the stance time (especially the single stance time) is too short, it may reflect limb pain or discomfort [26, 27].

The normal touchdown pattern was the knees almost extended and the heel touching the ground. In the swing phase, the maximum degree of knee flexion promotes the realisation of the maximum step length. The pre-TKA touchdown pattern was knee flexion, and almost the entire sole touched the ground. In the swing phase, a smaller knee flexion angle reduced the vertical distance of the heel to the ground, as well as the step length. These gait changes buffer the shock of heel touching the ground, thereby reducing pain [28]. While significantly improved, the patients’ gait spatiotemporal and knee joint motion parameters 1-year post-TKA were still significantly lower than that of the controls.

In the stance phase, the body’s centre of gravity goes through the process of behind the knee (accelerated decline), through the knee (accelerate to rise), and in front of the knee (re-accelerated descent), resulting in an m-shaped plantar pressure curve [29]. A double-peak ‘m’ shaped plantar pressure curve is essential for human articular cartilage nutrition; since articular cartilage has no blood vessels or lymphatic vessels and relies on the synovial fluid for nutrition and metabolism, changes in pressure maintain cartilage metabolism. Peak of the plantar pressure curve is equivalent to squeezing the articular cartilage, discharging metabolites. The trough of the plantar pressure curve is equivalent to releasing the pressure, allowing the nutrients to be absorbed; in the second peak, the metabolites will be re-discharged [30]. For patients who underwent TKA, the plantar pressure curve was not a typical double-peak ‘m’ shape, which cannot effectively squeeze, relax, or recompress the articular cartilage and is not conducive to the metabolism of articular cartilage.

Having gait balance and symmetry — including weight-bearing and activities of the lower limbs — is important for safe movement. One year after TKA, patients’ weight-bearing balance and walking stability significantly improved; however, these remained worse than in the control group. Long-term unbalanced weight-bearing inevitably causes excessive wear and reduces the service life of the prosthesis [31, 32]. Likewise, asymmetric step length and time pose a greater risk of falls in patients undergoing TKA [33, 34].

WOMAC scores are often used to assess knee joint function owing to their high internal consistency, cost-effectiveness, and ease of administration. This study confirmed a significant correlation and consistency between the WOMAC score and gait analysis for assessing knee function pre-TKA. However, there are still large gaps in the evaluation of knee joint function 1 year after TKA. WOMAC scores may not fully capture limitations in patient gait performance as they are influenced by patient experience and confidence in their abilities [35, 36]. Patients’ perceptions of functional recovery after TKA may be influenced by functional difficulties and pain levels pre-TKA; thus, patients are more likely to overestimate their ability after TKA when pain levels are substantially reduced [37, 38]. This study confirmed a significant positive correlation between pain and daily living function sub-items of the WOMAC score before and after TKA, consistent with other reports. In addition, patients with KOA often do not have a thorough understanding of TKA and thus have lower expectations. For patients with KOA who experienced significant pain before TKA, pain relief was the only purpose of TKA surgery. One year after TKA, the patients were relieved of pain and gained a certain degree of mobility; this exceeded their subjective expectations and led to overestimation of the effect of the surgery.

Although patient self-report satisfaction is critical post-TKA, objective gait deficits cannot be ignored. One-year post-TKA, patients’ gait function, although significantly improved, was still significantly lower than that of healthy, age-matched controls. This persistent gait function deficit predisposes patients to future disabilities with increasing age; therefore, attention should be paid to rehabilitating patients with KOA in all aspects.

Our study had some limitations; first, the post-TKA follow-up was performed at a single time point, which is not conducive to determining the correlation between WOMAC and gait analysis on a time axis. In the future, intelligent, wearable, portable gait devices should be developed to dynamically monitor patient changes. Second, the number of cases included in this study is relatively small, which may introduce certain bias to the research conclusion.

## Conclusions

The WOMAC scale considers patient perceptions of recovery, whereas gait analysis allows a more objective functional capacity evaluation. Both sets of tools provide different and complementary information and should be combined to analyse outcomes after TKA.

### Electronic supplementary material

Below is the link to the electronic supplementary material.

Supplementary Material 1

Supplementary Material 2

Supplementary Material 3

## Data Availability

The datasets used and/or analysed during the current study are available from the corresponding author on reasonable request (email: zhaoyu20806@163.com).

## Abbreviations

KOA: Knee osteoarthritis
TKA: Total knee arthroplasty
WOMAC: Western Ontario and McMaster University Osteoarthritis Index
