# Supplementary material for: Deficient gait function despite effect index of the Western Ontario and McMaster university osteoarthritis index score considered cured one year after bilateral total knee arthroplasty
Source: BMC Musculoskelet Disord. 2024 Mar 23;25:230. doi: 10.1186/s12891-024-07348-7 (PMC10960387; doi:10.1186/s12891-024-07348-7)
Supplement: Supplementary file 1 — Supplementary Material 1 [file 12891_2024_7348_MOESM1_ESM.docx]

**Deficient gait function despite effect index from the Western Ontario and McMaster University Osteoarthritis Index score considered cured one year after bilateral total knee arthroplasty**

**Supplementary Table 1 Pearson correlation analysis of WOMAC score and gait analysis in the experimental group after TKA (n=25)**

|  | Pain | | Stiffness | | Daily living function | | Full score | |
| --- | --- | --- | --- | --- | --- | --- | --- | --- |
|  | r | p | r | p | r | p | r | p |
| Velocity | -0.31 | 0.135 | 0.22 | 0.302 | -0.28 | 0.168 | -0.35 | 0.087 |
| Cadence | -0.04 | 0.848 | 0.17 | 0.416 | -0.05 | 0.830 | 0.003 | 0.989 |
| Step length | 0.28 | 0.184 | 0.29 | 0.153 | 0.16 | 0.440 | 0.1 | 0.663 |
| Stride length | 0.23 | 0.276 | 0.351 | 0.086 | 0.1 | 0.634 | 0.09 | 0.669 |
| Step time | 0.05 | 0.828 | 0.27 | 0.19 | 0.01 | 0.95 | -0.001 | 0.99 |
| Gait cycle | -0.15 | 0.486 | -0.27 | 0.189 | -0.17 | 0.417 | -0.24 | 0.257 |
| Total stance time | 0.06 | 0.789 | -0.08 | 0.706 | 0.15 | 0.463 | 0.10 | 0.634 |
| Double stance time | -0.17 | 0.411 | 0.14 | 0.503 | 0.14 | 0.498 | 0.08 | 0.709 |
| Single stance time | -0.09 | 0.640 | 0.002 | 0.993 | -0.13 | 0.535 | -0.12 | 0.571 |

**Supplementary Fig1**


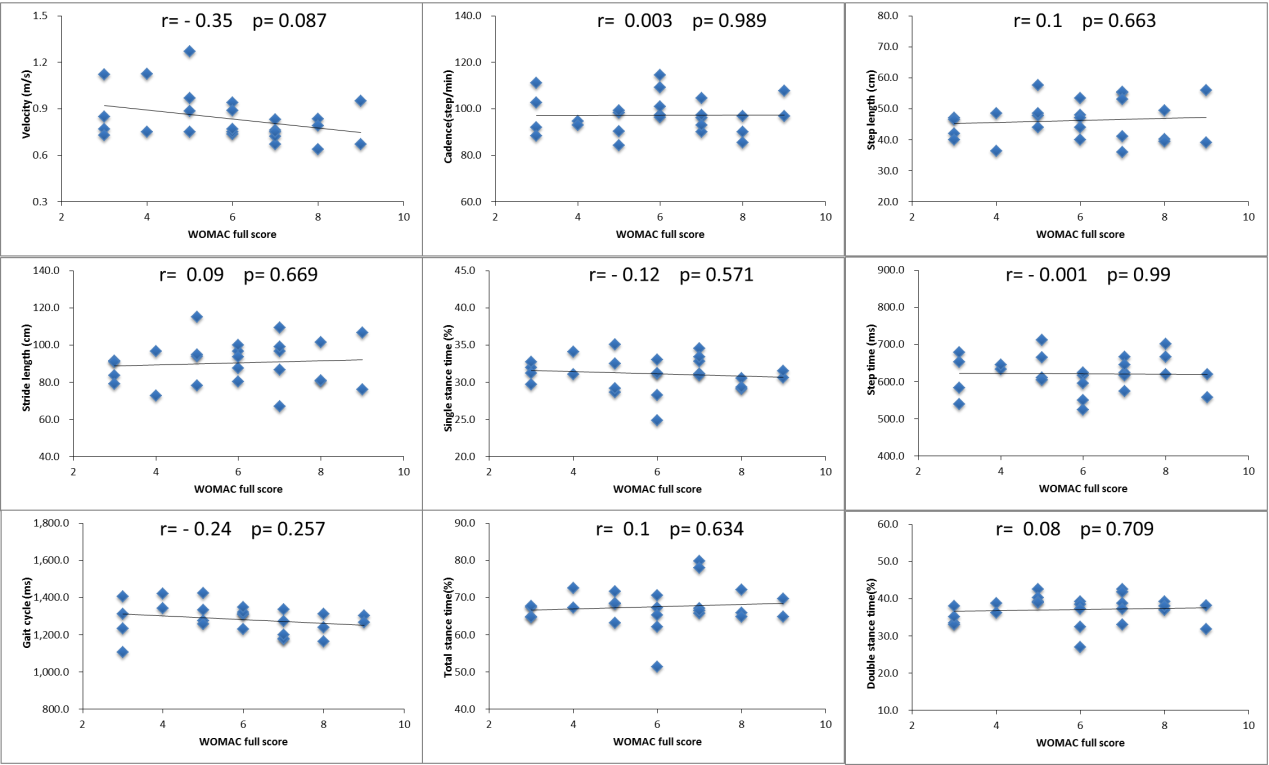


**Supplementary Fig1** Pearson correlation analysis of WOMAC full score and gait analysis in the experimental group after TKA. There was no significant correlation between the WOMAC full score and gait analysis parameters 1 year after TKA. (P>0.05, n=25)
