# Supplementary material for: Deficient gait function despite effect index of the Western Ontario and McMaster university osteoarthritis index score considered cured one year after bilateral total knee arthroplasty
Source: BMC Musculoskelet Disord. 2024 Mar 23;25:230. doi: 10.1186/s12891-024-07348-7 (PMC10960387; doi:10.1186/s12891-024-07348-7)
Supplement: Supplementary file 3 — Supplementary Material 3 [file 12891_2024_7348_MOESM3_ESM.pdf]

BioMed Central

Consent form

I ....Youchang Zhang.. [Name] give my consent for information about myself/my child or ward/my relative (circle as appropriate) to be published in ..BMC Musculoskeletal Disorders. Manuscript ID: 2d643207-c959-4f43-a5bd
-e77a1daa1748 V.2.0 ...... corresponding author: Yu Zhao..
[Name of journal, manuscript number and corresponding author].

I understand that the information will be published without my/my child or ward's/my relative's (circle as appropriate) name attached, but that full anonymity cannot be guaranteed.
I understand that the text and any pictures or videos published in the article will be freely available on the internet and may be seen by the general public. The pictures, videos and text may also appear on other websites or in print, may be translated into other languages or used for commercial purposes.
I have been offered the opportunity to read the manuscript.

Signing this consent form does not remove my rights to privacy.

Name....Youchang Zhang........
Date.....2024.2.18..............
Signed.....Youchang Zhang.......
Author name..Ruipeng Zhao, Xiaochun Wei, Shuai Hu, Yixuan Zhang, Hongru Wu, Pengcui Li, Yu Zhao.
Date...2024.2.18.................
Signed. Ruipeng Zhao, Xiaochun Wei, Shuai Hu, Yixuan Zhang. Hongru Wu. Pengcui Li, Yu Zhao.

Please keep this consent form in the patient's case files. The manuscript reporting this patient's details should state that 'Written informed consent for publication of their clinical details and/or clinical images was obtained from the patient/parent/guardian/ relative of the patient. A copy of the consent form is available for review by the Editor of this journal.

BioMed Central

Consent form

I ...Wu Xiao Wang.. [Name] give my consent for information about myself/my child or ward/my relative (circle as appropriate) to be published in BMC Musculoskeletal Disorders Manuscript ID: 2d643207-c959-4f45-a5bd-e71d1daa1748 V2.2 ...... Corresponding author ... Yu zhao...................................

[Name of journal, manuscript number and corresponding author].

I understand that the information will be published without my/my child or ward's/my relative's (circle as appropriate) name attached, but that full anonymity cannot be guaranteed.

I understand that the text and any pictures or videos published in the article will be freely available on the internet and may be seen by the general public. The pictures, videos and text may also appear on other websites or in print, may be translated into other languages or used for commercial purposes.

I have been offered the opportunity to read the manuscript.

Signing this consent form does not remove my rights to privacy.

Name.....Wu Xiao Wang.........

Date......2024.2.19.............

Signed....Wu Xiao Wang.........

Author name. Ruipeng zhao. Xiaochun wei, shuai Hu. Yixuan zhang. Hongru Wu. Pengcui Li. Yu zhao

Date...2024.2.19...............

Signed. Ruipeng zhao. Xiaochun wei, shuai Hu. Yixuan zhang. Hongru We. Pengcu Li. Yu zhao

Please keep this consent form in the patient's case files. The manuscript reporting this patient's details should state that 'Written informed consent for publication of their clinical details and/or clinical images was obtained from the patient/parent/guardian/ relative of the patient. A copy of the consent form is available for review by the Editor of this journal.
